# The relation between color and spatial structure for interpreting colormap data visualizations

**DOI:** 10.1167/jov.20.12.7

**Published:** 2020-11-17

**Authors:** Shannon C. Sibrel, Ragini Rathore, Laurent Lessard, Karen B. Schloss

**Affiliations:** Department of Psychology, Wisconsin Institute for Discovery, University of Wisconsin–Madison, Madison, WI, USA; Wisconsin Institute for Discovery, University of Wisconsin–Madison, Madison, WI, USA; Department of Computer Sciences, University of Wisconsin–Madison, Madison, WI, USA; Department of Mechanical and Industrial Engineering, Northeastern University, Boston, MA, USA

**Keywords:** visual reasoning, information visualization, configural processing, perceptual organization, color cognition

## Abstract

Interpreting colormap visualizations requires determining how dimensions of color in visualizations map onto quantities in data. People have color-based biases that influence their interpretations of colormaps, such as a dark-is-more bias—darker colors map to larger quantities. Previous studies of color-based biases focused on colormaps with weak data spatial structure, but color-based biases may not generalize to colormaps with strong data spatial structure, like “hotspots” typically found in weather maps and neuroimaging brain maps. There may be a hotspot-is-more bias to infer that colors within hotspots represent larger quantities, which may override the dark-is-more bias. We tested this possibility in four experiments. Participants saw colormaps with hotspots and a legend that specified the color-quantity mapping. Their task was to indicate which side of the colormap depicted larger quantities (left/right). We varied whether the legend specified dark-more mapping or light-more mapping across trials and operationalized a dark-is-more bias as faster response time (RT) when the legend specified dark-more mapping. Experiment 1 demonstrated robust evidence for the dark-is-more bias, without evidence for a hotspot-is-more bias. Experiments 2 to 4 suggest that a hotspot-is-more bias becomes relevant when hotspots are a statistically reliable cue to “more” (i.e., the locus of larger quantities) and when hotspots are more perceptually pronounced. Yet, comparing conditions in which the hotspots were “more,” RTs were always faster for dark hotspots than light hotspots. Thus, in the presence of strong spatial cues to the locus of larger quantities, color-based biases still influenced interpretations of colormap data visualizations.

## Introduction

To interpret meaning from information visualizations, people leverage core aspects of visual processing that extend over lower-level perceptual discrimination (e.g., Healey, 1996; Stone, Szafir, & Setlur, 2014; Szafir, 2017), mid-level perceptual organization (e.g., Gramazio, Schloss, & Laidlaw, 2014; Haroz & Whitney, 2012; Nothelfer, Gleicher, & Franconeri, 2017; Rosenholtz, Twarog, Schinkel-Bielefeld, & Wattenberg, 2009), and higher-level visual reasoning (e.g., Gattis & Holyoak, 1996; Haroz, Kosara, & Franconeri, 2015; Schloss, Gramazio, Silverman, Parker, & Wang, 2019; Zacks & Tversky, 1999). Lower-level visual processing is essential for perceiving input from information visualizations, and mid-level processing is key for organizing visual input, but it is visual reasoning that enables people to connect perceptual properties (e.g., gradations of color) to abstract concepts represented in visualizations (e.g., increased brain activity represented in neuroimaging). Researchers have established that visual reasoning is easier for observers when there is a strong correspondence between the properties of visual features in visualizations and concepts that those visual features represent (Giardino & Greenberg, 2014; Kosslyn, 2006; Lin, Fortuna, Kulkarni, Stone, & Heer, 2013; Norman, 1988, 2013; Schloss et al., 2019; Schloss, Lessard, Walmsley, & Foley, 2018; Tversky, 2011; Tversky, Morrison, & Betrancourt, 2002; Zacks & Tversky, 1999). However, what factors determine strong correspondence between perceptual and conceptual properties is still an open question.

We approach this question by studying colormap data visualizations, like those found on weather maps, neuroimaging brain maps, spectrograms, correlation matrices, and gene expression matrices. Colormaps use color variation as a channel for visual communication (Bertin, 1983; Brewer, 1994; Brewer, Hatchard, & Harrower, 2003; Bujack et al., 2018; Harrower & Brewer, 2003; Rogowitz & Treinish, 1996; Roth, Woodruff, & Johnson, 2010; Ware, 1988). Colormaps are produced by mapping colors within a color scale (e.g., gradations from white to black) onto quantities in a data set and then representing those quantities within a set of spatial coordinates (Hegarty, 2011). Studying colormaps provides an interesting path to understanding how people infer mappings between visual features and concepts because there are multiple levels of correspondence to consider.

The first level of correspondence concerns the properties of perceptual features and concepts. Colormaps map continuous perceptual dimensions (e.g., variation in lightness) to continuous conceptual dimensions (e.g., variations in quantity). This sort of structural preservation is considered a *natural* correspondence (Giardino & Greenberg, 2014; Palmer, 1978). As such, much of the research on colormaps has focused on understanding which perceptual properties help make color scales appear continuous and uniform, even when colors are spatially scattered within colormap visualizations (Borland & Taylor, 2007; Brewer, 1994, 1997; Liu & Heer, 2018; Moreland, 2009; Reda, Nalawade, & Ansah-Koi, 2018; Rogowitz & Treinish, 1998; Silva, Santos, & Madeira, 2011; Zhou & Hansen, 2016; for a review, see Bujack et al., 2018).

The second level of correspondence concerns the semantic mapping between perceptual features and concepts. For example, a given color scale can be mapped such that the darker colors correspond to larger quantities or smaller quantities within a data set. One might imagine that the direction of this mapping is inconsequential if specified by a legend, but colormaps in published articles do not always have legends (Christen et al., 2013; Schott, 2010), and when they do, the direction of assignment matters. People are faster at interpreting colormaps when mapping specified by the legend (*encoded mapping*) matches their expectations (*inferred mapping*) (Schloss et al., 2019). Empirical work has begun to establish how inferred mappings are influenced by properties of colors in colormaps (Cuff, 1973; McGranaghan, 1989; Schloss et al., 2019), but questions remain about how those color-based biases are influenced by other properties of colormaps, such as spatial structure. In the following sections, we summarize prior work on inferred color-quantity mappings and raise open questions about spatial structure that motivated the present study.

## Inferred mappings for colormap data visualizations

Inferred mappings are influenced by at least two biases—a *dark-is-more bias* that darker colors map to larger quantities (Cuff, 1973; McGranaghan, 1989; Palsky, 1999; Robinson, 1952; Schloss et al., 2019) and an *opaque-is-more bias* that more opaque colors map to larger quantities (Schloss et al., 2019). Initial studies on the dark-is-more bias asked participants to interpret colormaps without legends and found that participants reported that regions of darker colors indicated larger quantities (Cuff, 1973; McGranaghan, 1989).

**Figure 1.**
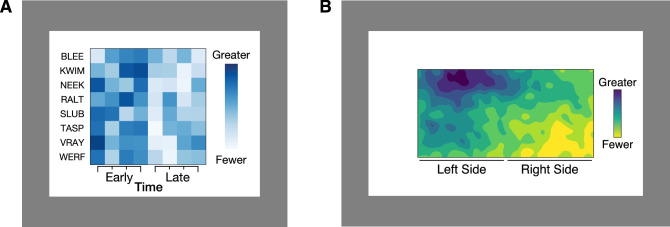
(A) Example trial from Schloss et al. (2019). Participants saw colormaps with legends and indicated whether there were more alien animal sightings early or late in the day. (B) Example trial in the present study. Participants saw colormaps with legends and indicated whether there were more alien animal sightings on the left or right side of the map.

In a different paradigm, Schloss et al. (2019) asked participants to interpret colormaps that included legends, so there was an objectively correct answer. We explain this paradigm in detail because it is the basis for the present study. The colormaps represented fictitious alien animal sightings in different regions of the planet Sparl, where the x-axis represented time and the y-axis represented different categories of alien animals in a grid structure (Figure 1A). The colors were biased so one side was visibly darker and the other lighter, balanced across trials. The colormaps appeared on light or dark backgrounds. On half of the trials, the legend specified that darker colors mapped to larger quantities (dark-more encoded mapping, as in Figure 1A), and on the other half of the trials, the legend specified lighter colors mapped to larger quantities (light-more encoded mapping). The encoded mapping was manipulated by balancing the orientation of the color scale in the legend (darker endpoint at the top or the bottom) and the positions of the text in the legend (“Greater” at the top or the bottom). Using the legend, participants indicated whether there were more animal sightings early (left half of colormap) or late (right half of colormap) in the day. A dark-is-more bias was operationalized as faster response times (RTs) when the legend specified dark-more encoding than when it specified light-more encoding.

Results showed a robust dark-is-more bias when colormaps did not appear to vary in opacity on their given background color. However, the opaque-is-more bias came into play when colors did appear to vary in opacity (see Roth et al., 2010, for techniques for constructing colormaps that appear to vary in opacity [“value-by-alpha” maps]). The opaque-is-more bias and dark-is-more bias work together on light backgrounds (darker colors appear more opaque), but they conflict on dark backgrounds (darker colors appear less opaque). When they conflict, the opaque-is-more bias can cancel or even override the dark-is-more bias (Schloss et al., 2019). If colormaps do not appear to vary in opacity, the background color has little effect, and the dark-is-more bias dominates inferred mappings (McGranaghan, 1989; Schloss et al., 2019).

## The role of spatial structure?

Previous studies on inferred mappings for colormaps focused on mappings between colors and quantities, with less consideration of the spatial structure within colormap visualizations. In colormaps, color variation is overlaid on some visual-spatial structure (e.g., a geographic map or a brain map) to signal magnitude in a third dimension of a data set (e.g., wind speeds or neural activity), which is not represented in the visual-spatial structure (Hegarty, 2011). We call this type of visual-spatial structure *background spatial structure* because it refers to the background on which the colormap is overlaid. Studies on the dark-is-more bias used different types of background spatial structure, including a partial map of the United States (McGranaghan, 1989) and a grid (Schloss et al., 2019), which suggests that the dark-is-more bias may be robust to background spatial structure.

Background spatial structure is distinct from what we call *data spatial structure,* which is the pattern of magnitudes (third dimension) across the x and y coordinates of the data set. Some types of data sets tend to have strong spatial structure, such as “hotspots,” with the largest magnitudes concentrated at center points (e.g., weather patterns or neural activity) (Schott, 2010). Other types of data sets tend to have weaker spatial structure, with high and low values scattered throughout the data set (e.g., election results).

Schott (2010) suggested that for colormaps with hotspots, it is the spatial distribution of colored areas that matters, not the colors themselves. Referring to neuroimaging data, Schott (2010) posited, “I suggest, however, that surprisingly, the “hottest” area is revealed not necessarily by the individual colors themselves but by their “location”. Surely, we tend to assume the center of the area to be the “hottest” (or “coldest”)— in the same way that we assume the center of a set of concentric rings on a weather map betokens the eye of the storm?” (p. 516). Thus, people may have a space-based, hotspot-is-more bias that overrides color-based biases like the dark-is-more bias. Prior studies have primarily focused on colormaps with weak data spatial structure (McGranaghan, 1989; Schloss et al., 2019), and it is unknown how, if at all, the results will generalize to colormaps with hotspots, typically found in weather maps and neuroimaging. Although Cuff's (1973) colormaps did have hotspots, there were comparable amounts of light and dark hotspots within a given colormap, so the dark-is-more bias and potential hotspot-is-more bias were never under direct conflict.

In the present study, we investigated how the presence of hotspots influenced the dark-is-more bias for interpreting colormap data visualizations. We had several reasons to suspect that hotspot structure might override the dark-is-more bias. First, hotspots reflect inherent structure in the data, whereas coloration is a surface property that can be arbitrarily applied to spatial structure. Second, hotspots may be a familiar cue to “more,” as they tend to signal larger quantities in common visualizations, like weather maps (Schott, 2010). Third, people tend to prioritize shape over surface properties like color or texture when learning to categorize objects (Landau, Smith, & Jones, 1988), which may reflect a general bias to prioritize spatial structure (although see Macario [1991] and Colunga & Smith [2005] for exceptions). Fourth, data visualization researchers have argued that spatial factors are more effective than color for comparisons in graphs (Cleveland & McGill, 1984) (although see Albers, Correll, & Gleicher [2014] for exceptions). Finally, hotspots may be perceived as more “figural” in terms of figure-ground organization because they are relatively small and surrounded (Rubin, 1958). It is not immediately obvious why people might infer that figural status represents larger quantities, but such an account might involve the figure comprising more “stuff” compared to the “stuffless” background that extends behind it.

**Figure 2.**
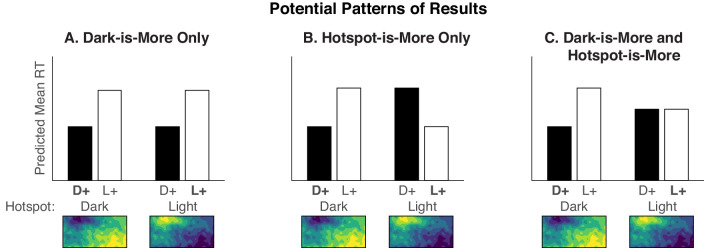
Predicted RTs for dark-more encoding (D+, black bars) and light-more encoding (L+, white bars) for dark and light hotspots (x-axis), depending on if there is (A) only a dark-is-more bias, (B) only a hotspot-is-more bias, or (C) a combination of both a dark-is-more and hotspot-is-more bias. Conditions in which the colors in the hotspot map to larger quantities are indicated by bold text (i.e., **D**+ when the hotspots are dark and **L**+ when hotspots are light).

In the present study, we operationalized the dark-is-more bias as faster RTs when the legend specified dark-more encoding than when it specified light-more encoding (as in Schloss et al., 2019). We operationalized a potential hotspot-is-more bias as faster RTs when the legend specified that the colors in the hotspot mapped to larger quantities than when the colors outside the hotspot mapped to larger quantities. Figure 2 shows different potential patterns of results that would occur depending on whether RTs are determined by only a dark-is-more bias, only a hotspot-is-more bias, or both. If there is only a dark-is-more bias, RTs should be faster for dark-more encoding (D+), regardless of whether the hotspot is dark or light (Figure 2A). If there is only a hotspot-is-more bias, RTs should be faster for dark-more encoding for dark hotspots and light-more encoding for light hotspots (Figure 2B). Finally, if there is both a dark-is-more bias and a hotspot-is-more bias, RTs should be faster for dark-more encoded mappings when hotspots are dark, and this difference should be reduced, eliminated, or even slightly reversed when hotspots are light (Figure 2C).

In the following experiments, we first tested how the dark-is-more bias and a potential hotspot-is-more bias contributed to interpretations of colormaps, using a paradigm similar to Schloss et al. (2019) (Experiment 1). We then studied the role of the hotspot when it was a more reliable cue (Experiment 2) and when it was more visually pronounced through increasing lightness contrast (Experiment 3) and decreasing noise in the underlying data used to generate the colormaps (Experiment 4). We found that the dark-is-more bias was far more robust than expected. It took making the hotspot a reliable cue and strongly visually pronounced for the hotspot-is-more bias to override the dark-is-more bias.

## Experiment 1

**Figure 3.**
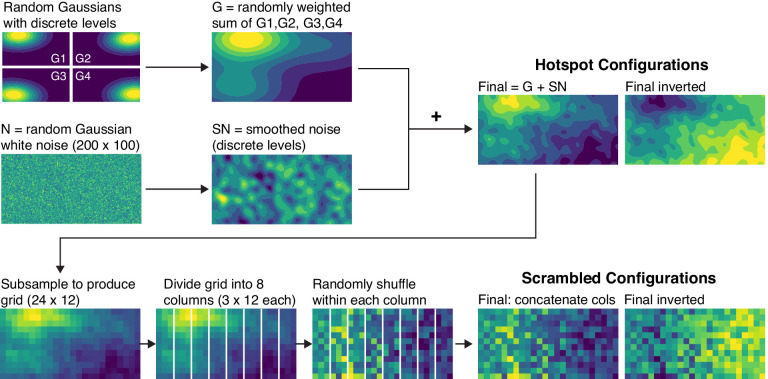
Illustration of the process used to generate colormaps with hotspot configurations and scrambled configurations (see the description in the text for details).

In Experiment 1, we tested for the dark-is-more bias in colormaps that had strong data spatial structure (“hotspot” configurations) and weak data spatial structure (“scrambled” configurations); see Figure 3. Our experiment paradigm was adapted from Schloss et al. (2019), but we modified the colormap design so the colormaps represented fictitious alien animal sightings in different regions of the planet Sparl. The x-axis and y-axis represented horizontal and vertical coordinates on the planet (Figure 1B) rather than time and category of animals (Figure 1A). This change enabled us to produce continuous underlying data sets with hotspot spatial structure. We used fictitious data about a fictitious world to avoid potential knowledge effects that could arise from testing familiar data sets (e.g., weather maps). We tested colormaps that should not appear to vary in opacity (Schloss et al., 2019), so we focused on the dark-is-more bias and not the opaque-is-more bias. The scrambled configurations were similar to the stimuli in Schloss et al. (2019), which enabled us to replicate prior results.

### Methods

#### Participants

The participants were 68 undergraduates at the University of Wisconsin–Madison (mean age = 19.91, 34 females, 34 males), who received extra credit in their introductory psychology course for their participation. We excluded six participants from the analysis because their overall accuracy was less than 90% (criteria determined a priori, following the protocol in Schloss et al., 2019), and we excluded two other participants because they did not complete the experiment. Participants were divided into two groups, balancing whether they completed hotspot colormap trials first and scrambled colormap trials second (*n* = 30) or the opposite order (*n* = 30). This sample size was chosen to match Experiment 2 of Schloss et al. (2019). All participants had normal color vision (tested with HRR Pseudoisochromatic Plates; Hardy, Rand, Rittler, Neitz, & Bailey, 2002) and gave informed consent. The University of Wisconsin–Madison Institutional Review Board approved the experimental protocol for this and all subsequent experiments.

#### Design and displays

To establish terminology, we refer to colormap data visualizations as *colormaps*, the data set used to generate the colormap as *underlying data*, and the sequence of colors used to assign color to the underlying data as the *color scale* (sometimes referred to as *color ramps* elsewhere; Smart, Wu, & Szafir, 2020). The color scale for each colormap is displayed in the legend with *legend text* that specifies the encoded mapping between endpoints of the color scale and the concepts “Greater” and “Fewer.”

Figure 1B shows the layout of the test displays. The colormap (500 × 250 pixels, 13.5 × 6.8 cm) was centered on the screen and surrounded by a black outline (1 pixel thick). The color scale (21 × 135 pixels, .5 × 3.8 cm), positioned 65 pixels (1.4 cm) to the right of the right edge of the colormap, was also surrounded by a black outline (1 pixel thick). There were two line segments (240 × 2 pixels) below the colormap (16 pixels below the bottom edge) that marked the left and right sides of the map, with the label “Left Side” centered below the left line segment and “Right Side” below the right line segment. This was done to help participants parse the left and right sides while doing the task. The colormap and legend appeared on a rectangular white background (450 × 780 pixels, 21 × 12.1 cm), centered behind the colormap. The white was the native white of the monitor (RGB = [255, 255, 255]). The surrounding background of the monitor was a neutral gray (RGB = [128, 128, 128]). The color scales were specified using RGB coordinates for the Autumn color scale (from MATLAB, Natick, MA, USA) and the Viridis color scale (from Matplotlib).^1^ The colormaps were generated in MATLAB (see below), and the rest of the layout was produced and displayed using Presentation (www.neurobs.com). Displays were shown in a dark room using a 24.1-in. ASUS (Taipai, Taiwan) ProArt PA249Q monitor (1,920 × 1,200 resolution) from a viewing distance of approximately 60 cm.

**Figure 4.**
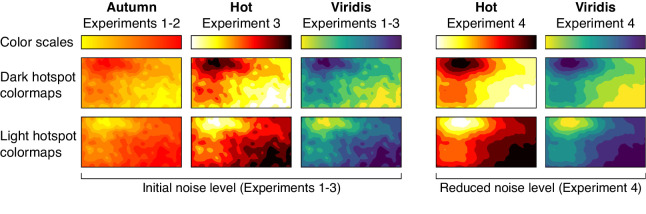
The top row shows the three color scales tested in this study: Autumn (Experiments 1–2), Hot (Experiments 3–4), and Viridis (Experiments 1–4). These color scales are applied to the same underlying data sets to produce colormaps with dark hotspots (middle row) and light hotspots (bottom row). The three left columns show colormaps constructed from underlying data sets with the initial noise level used in Experiments 1 to 3, and the two right columns show colormaps constructed from underlying data sets with reduced noise used in Experiment 4.

Figure 3 shows how we constructed the hotspot and scrambled colormaps (code is available at https://github.com/SchlossVRL/colormaps). To produce the hotspot colormaps, we first generated contour maps for four random Gaussian distributions positioned in each of the four quadrants of the image (G1, G2, G3, and G4) and quantized to nine discrete colors. The image was 500 × 250 pixels. We then computed G as a random linear combination of G1–G4. Next, we generated a distribution of random Gaussian white noise (N), smoothed and quantized it (SN), and added SN to G to produce a final colormap. By default, these colormaps had light hotspots, and we inverted them to produce corresponding colormaps with dark hotspots. The Gaussian in the top left corner was assigned a random mean that was larger than that in the other three quadrants, thus producing a hotspot in the top left corner. To balance the hotspot position, we first reflected half of these images across the horizontal axis. We then duplicated all of these images and reflected them across the vertical axis, such that all colormaps were left/right balanced across trials. We also applied two different types of color scales to the data set, “Autumn” (yellow to red; MATLAB) and “Viridis” (yellow to green to blue; Matplotlib), to ensure that any result we observed were not specific to a particular set of colors (Figure 4). Thus, a single underlying data set generated eight hotspot colormap images from the combination of 2 hotspot lightnesses (light or dark)  ×  2 hotspot sides (left or right) × 2 color scales (Autumn or Viridis).

To produce the scrambled colormaps, we started with the data sets in the final hotspot configurations and scrambled the data. First, we subsampled the image to produce a 24 × 12 grid. Next, we divided the grid into eight 3 × 12 columns, shuffled the cells within each column to weaken the spatial structure, and concatenated the columns to produce the final scrambled configuration.

Using this procedure, we generated 100 sets of colormaps with different underlying data sets (each set included color scale inversion, left/right reflection, and Viridis and Autumn color scales for hotspot and scrambled configurations). The random variation in our procedure sometimes resulted in hotspot configurations in which hotspots looked equally concentrated on both sides or scrambled configurations in which lightness appeared similar on both sides. In an initial pilot experiment, we simply picked the first 20 data sets generated by our algorithm, but there was a concern that the hotspot location was not clear in some of the hotspot configurations, and the darker side was not clear in some of the scrambled configurations. Therefore, we examined the hotspot and scrambled configurations produced by each underlying data set and selected the first 20 data sets for which hotspots were distinctly concentrated on one side for hotspot configurations, and one side was distinctly darker for scrambled configurations. In total, we tested 320 colormap images: 2 configurations (hotspot or scrambled) × 2 hotspot lightnesses (dark or light) × 2 hotspot sides (left or right) × 2 color scales (Viridis or Autumn) × 20 underlying data sets.

Each of the 320 colormap images appeared four times to accommodate four legend conditions, as in Schloss at al. (2019). This included 2 color scale orientations (dark-top or dark-bottom) × 2 legend text locations (“Greater”-top or “Greater”-bottom). Thus, the legend specified dark-more encoding when “Greater” and dark were both at the top or both at the bottom of the legend and light-more encoding when “Greater” and light were both at the top or the bottom of the legend. This design ensured that participants had to read the legend on every trial in order to accurately interpret the colormap visualization. The combination of 320 colormap images × 4 legend conditions produced a total of 1,280 trials, within subject. Trials were blocked by colormap configuration (hotspot/scrambled), and block order was balanced between subjects.

#### Procedure

Participants were told that they would see colormaps representing alien animal sightings on a distant planet, Sparl. Each map would have a legend, and sometimes greater amounts of sightings would be represented at the top of the legend and sometimes greater would be represented at the bottom. They would see many of these colormaps showing data from different locations on the planet. On each trial, their task would be to look at the colormap and the legend and decide whether there were more sightings in regions on the left or right side of the map and to respond by pressing the corresponding arrow key. They were asked to be as fast as possible while maintaining their accuracy. They were told that they would hear a tone each time they made an error, and they would be notified of their percent accuracy periodically.

Prior to the start of the experiment, participants were shown examples of colormaps to get an overview of the types of stimuli they would encounter. Then, they completed 20 practice trials that were randomly selected from the set of all possible conditions. During the practices and in the experiment, each trial began with a 500-ms blank gray screen followed by an experimental display with a colormap and legend. The colormap and legend remained on the screen until the participants responded. During the experiment, the colormaps were presented using a blocked randomized design: All 32 possible conditions were presented (2 hotspot lightnesses [dark or light] × 2 hotspot sides [left or right] × 2 color scales [Viridis or Autumn] ×  4 legend conditions) in a random order before going on to the next block. Within each block, we randomized which colormap images (given their underlying data sets) were assigned to each of the 32 conditions. There were 20 of these blocks of 32 trials to accommodate all 20 underlying data sets used to generate the colormaps. Participants were notified of their accuracy after each set of 20 trials, and they were informed when they completed 25%, 50%, and 75% of the trials.

### Results and discussion

We prepared RTs for analysis for each participant by eliminating trials with errors, calculating the mean and standard deviation across all remaining trials, and pruning trials with RTs that were ± 2 standard deviations from the mean. We then calculated the mean RT across all remaining trials out of 20 for each of the experimental conditions and averaged over colormaps that were left/right reflections of each other. The code used to process the data and the data sets for all experiments can be found at https://github.com/SchlossVRL/colormaps.

In the following sections, we first present results for the hotspot configurations, followed by results for the scrambled configurations. In the main text, we focus on effects of encoded mapping and hotspot lightness to address our main research questions and effects of color scale to test generalizability. Results involving legend text position and block order can be found in the Supplementary Material.

#### Hotspot configurations

**Figure 5.**
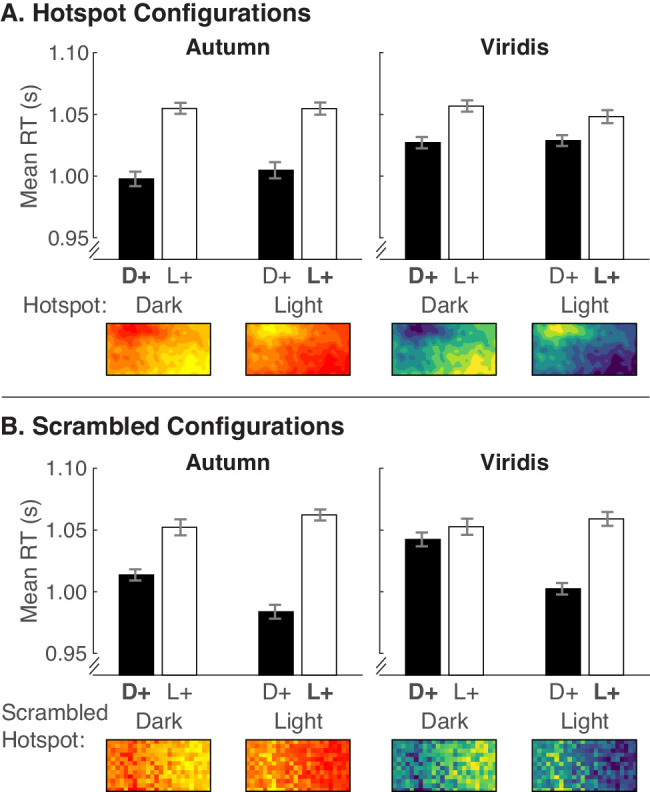
Mean RTs for the (A) hotspot configurations and (B) scrambled configurations from Experiment 1. Black bars represent dark-more encoding (D+) and white bars represent light-more encoding (L+). Bars are grouped according to hotspot lightness (or scrambled hotspot lightness) (x-axis). Data are separated by the Autumn and Viridis color scales. Conditions in which the colors in the hotspot map to larger quantities are indicated by bold text (i.e., **D**+ when the hotspots are dark and **L**+ when hotspots are light). Error bars represent standard errors of the means using the Cousineau (2005) adjustment to account for overall differences in RT at the subject level.

Figure 5A shows mean RTs for the hotspot configurations, plotted in the same manner as the predictions in Figure 2 (see Supplementary Figure S1 for data separated by legend text position). We analyzed the data using a mixed-design analysis of variance (ANOVA) with four within-subject factors (2 encoded mappings × 2 hotspot lightnesses × 2 color scales × 2 legend text positions) and one between-subject factor (2 block orders). Table 1 shows effects involving variables illustrated in Figure 5A, and Supplementary Table S1 shows the full set of results.

**Table 1.** Results of a mixed-design ANOVA comparing encoded mapping (Mapping) × hotspot lightness (HSLightness) × color scale (Colors) × legend text position (LegText) × testing order (Order) for hotspot configurations and scrambled configurations (Experiment 1). Degrees of freedom for all tests were (1, 58). **p < .01, ***p < .001. Results of the full analysis (including LegText and Order) are in Supplementary Table S1.

	Hotspot configurations			Scrambled configurations		
Source	*F*	*p*	$\eta_{p}^{2}$	*F*	*p*	$\eta_{p}^{2}$
Mapping	36.48	***	0.386	55.99	***	0.491
HSLightness	0.00	0.993	0.000	14.39	***	0.199
Colors	10.31	0.002**	0.151	9.46	0.003**	0.140
Mapping * HSLightness	1.96	0.167	0.033	36.34	***	0.385
Mapping * Colors	16.80	***	0.225	8.99	0.004**	0.134
HSLightness * Colors	1.27	0.264	0.021	1.17	0.284	0.020
Mapping * HSLightness * Colors	0.08	0.776	0.001	0.21	0.649	0.004

Addressing our main question, the pattern of RTs in Figure 5A resembles the dark-is-more bias only prediction (Figure 2A), with faster RTs for dark-more encoding, regardless of hotspot lightness. These observations are supported by a main effect of encoded mapping and no interaction between encoded mapping and hotspot lightness (Table 1).

Although this pattern was apparent for both Autumn and Viridis color scales, color scale did have an effect. RTs were overall faster for Autumn than Viridis, consistent with a prior report that RTs were fastest for Autumn (Schloss et al., 2019). This difference may be related to Autumn having less color variability than Viridis. There was also an interaction between color scale and encoded mapping, such that the difference between dark-more encoding and light-more encoding was greater for Autumn than Viridis. This interaction may too be explained by the reduced color variability in Autumn. Reduced color variability could make it especially easy to interpret colormaps when the encoded mapping matches the inferred mapping (e.g., encoded mapping aligns with the dark-is-more bias).

#### Scrambled configurations

We included scrambled configurations in this experiment because they were similar to the grid-like configurations in Schloss et al. (2019) (where robust a dark-is-more bias was found) and matched the statistics in the underlying data used to construct the hotspot configurations. We planned to use results from the scrambled configurations as a basis of comparison in case we observed widely different results for the hotspot configurations. As described above, the hotspot configuration results conformed with prior evidence for a dark-is-more bias, but we still discuss the scrambled configuration results here.

We analyzed RTs for the scrambled configurations in Figure 5B using the same mixed-design ANOVA as for the hotspot configurations, with four within-subject factors (2 encoded mappings × 2 hotspot lightnesses × 2 color scales × 2 legend text positions) and one between-subject factor (2 block orders); see Table 1. Recall that scrambling the underlying data within pairs of columns eliminated the hotspots but preserved the left/right differences in lightness of the colormaps. “Hotspot lightness” refers to the lightness of the hotspots before they were scrambled, so we call it “scrambled hotspot lightness” for the scrambled configurations hereafter.

Similar to the hotspot configurations in Figure 5A, Figure 5B shows a dark-is-more bias for scrambled configurations, with faster RTs for dark-more encoding than light-more encoding (main effect of encoded mapping in Table 1). However, there was an unexpected effect of scrambled hotspot lightness and an encoded mapping ×  scrambled hotspot lightness interaction. The effect of scrambled hotspot lightness revealed faster RTs when the scrambled hotspot was light, and the interaction revealed a greater difference between dark-more and light-more encoded mapping when the hotspot was light.

We had expected that scrambling the hotspots would result in no differences between dark hotspot and light hotspot images. However, in many of the scrambled images, the region previously containing the hotspot appeared to occupy less than half of the image. For example, if the hotspot was on the left and was light, the left ∼1/3 of the image appeared light biased, and the right ∼2/3 of the image appeared dark biased. Thus, effects concerning scrambled hotspot lightness may be related to perceived region size and point to a potential “larger-is-more” bias (i.e., inference that the colors in larger regions map to larger quantities). Yet, it is unclear why a region size bias would occur for the scrambled images and not the hotspot images, given that their region sizes were parallel. We did not design the stimuli to test for possible region size biases, and our discussion of these results is speculative. Future work is needed to understand these effects, but that is beyond the scope of the present study.

We also observed effects involving color scale, similar to results for the hotspot configurations. RTs were faster for Autumn than Viridis, and this difference was driven by faster RTs for Autumn than Viridis for dark-more encoding and little difference for light-more encoding (encoded mapping × color scale interaction). Again, these effects involving color scale could suggest that less color variability might lead to faster RTs when the encoded mapping matches the inferred mapping.

The key finding in Experiment 1 was a robust dark-is-more bias for hotspot configurations, without evidence for a hotspot-is-more bias. This result challenged Schott's (2010) prediction that hotspot spatial structure would dominate people's interpretations of colormaps. Surprised by this result, we considered possible explanations for why the hotspot had little effect. First, the hotspot was an unreliable cue to the locus of larger quantities within our experimental paradigm. On 50% of the trials, the encoded mapping specified that the color in the hotspot mapped to “Greater,” and on 50% of trials, it mapped to “Fewer.” These trial statistics may violate statistics of real-world visualizations. If hotspots tend to signal larger quantities in real-world visualizations as Schott (2010) suggested, participants might have learned to ignore the hotspot. We note, however, that the dark-is-more bias occurred even though encoded mapping was also an unreliable cue (i.e., on 50% of trials, there was dark-more encoding, and on 50%, there was light-more encoding). These results suggest that participants may be able to ignore the unreliable spatial cues but not unreliable color cues. Second, although we tried to make the hotspots visually pronounced in constructing our colormaps, it is possible that the participants did not detect the hotspot structure. We address both of these possibilities in Experiment 2.

## Experiment 2

In Experiment 2, we tested whether hotspots influenced the interpretations of colormaps if hotspots were a more reliable cue to the locus of larger quantities. We used the same basic paradigm as in Experiment 1 but altered the distribution of trials such that the lightness of the region within the hotspot mapped to larger quantities on 77% of the trials (as opposed to 50% in Experiment 1). We also included an additional hotspot localization task at the end of the experiment to check that participants could perceive hotspots in the colormap images.

### Methods

#### Participants

The participants were 35 undergraduates at the University of Wisconsin–Madison (mean age = 19.29, 16 females, 19 males) who participated for extra credit in their introductory psychology course. We analyzed data from 30 participants, excluding 3 whose overall accuracy was less than 90%, 1 who was color deficient, and 1 who did not complete the experiment. The rest of the participants had normal color vision, and all gave informed consent.

#### Design, displays, and procedure

All participants completed two tasks: the colormap interpretation task from Experiment 1, followed by a new, hotspot localization task. The design, displays, and procedure for the colormap interpretation task were the same as in Experiment 1 with two exceptions. First, the participants only completed trials for the hotspot configurations (not the scrambled configurations). Second, we altered the distribution of trials such that the hotspot lightness mapped to “more” on 77% of the trials and the surround lightness mapped to “more” on 23% of the trials, using the following procedure. Recall that in Experiment 1, there were 20 underlying data sets that each produced four colormap images for each color scale (2 hotspot lightnesses [dark or light] × 2 hotspot positions [left or right]). Each colormap image appeared four times so it could be paired with four different legend conditions: 2 encoded mappings (dark-more or light-more) × 2 legend text positions (Greater-high or Greater-low). In Experiment 2, we used this same design for 6 of the 20 underlying data sets, and we refer to these as the “balanced cue images.” For the other 14 underlying data sets, we only presented trials in which the hotspot was “more.” To do so, we paired dark hotspot images only with legends that had dark-more encoding and light hotspot images only with legends that had light-more encoding. For these colormap images, the spatial cue to the hotspot and the color-quantity mappings were redundant cues to the locus of larger quantities. We refer to these as the “hotspot-more images.” This design produced a total of 416 trials. The 20 practice trials were all hotspot-more trials to help build the expectation that the hotspot was a reliable cue at the start of the experiment.

In the hotspot localization task, participants saw each of the 160 hotspot colormap images four times, one at a time in a random order (640 trials). No legend was present. Participants were told that each colormap would contain a hotspot of concentric regions, similar to what they would see on a weather map showing the center of a storm. Their task would be to look at the colormap and indicate whether the hotspot appeared on the left or right side by pressing the corresponding arrow key. During the instructions, they were shown eight example colormaps and were asked to point to the side that had the hotspot. This was done to ensure that the participant understood the task. If they asked questions about where the hotspot was, the experimenter reminded them of the weather map example and told them to use their best judgment. The participants were notified when they completed 25%, 50%, and 75% of the trials.

### Results and discussion

We prepared RTs for analysis using the same procedure as in Experiment 1. One participant did not have any valid trials for one condition after pruning for errors and RT outliers, so we replaced that missing cell with that participant's mean RT for all the other conditions.

**Figure 6.**
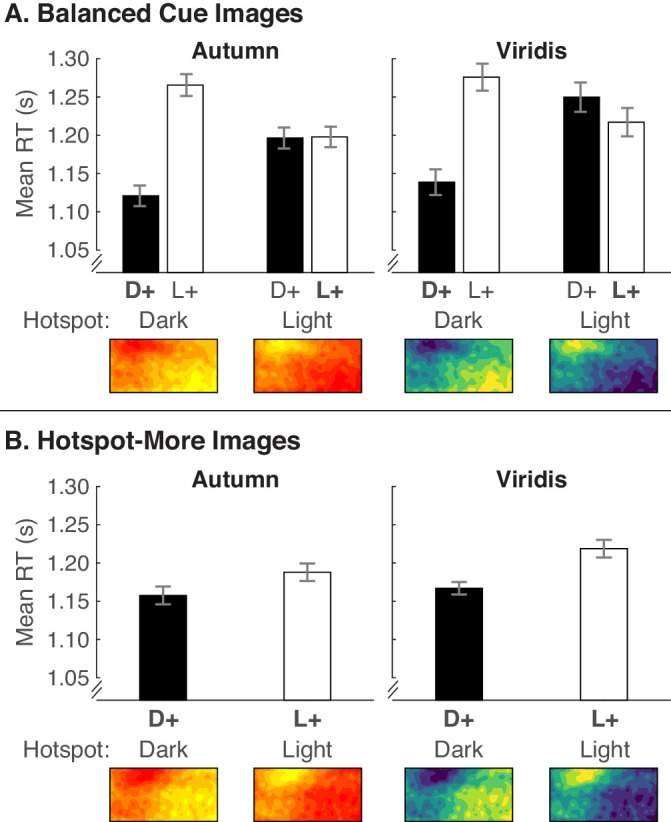
Mean RTs from Experiment 2 for (A) balanced cue images and (B) hotspot-more images, separated for dark-more encoding (D+, black bars) and light-more encoding (L+, white bars) depending on whether the hotspots were dark or light (x-axis). Data are separated by the Autumn and Viridis color scales. Conditions in which the colors in the hotspot map to larger quantities are indicated by bold text (i.e., **D**+ when the hotspots are dark and **L**+ when hotspots are light). Error bars represent standard errors of the means using the Cousineau (2005) adjustment to account for overall differences in RT at the subject level.

Figure 6A shows the mean RTs for the balanced cue images, which appeared in equal amounts of trials with legends that specified the color in the hotspot mapped to “Greater” versus “Fewer.” Figure 6B shows mean RTs for the hotspot-more images, which only appeared with legends that specified that the color in the hotspot mapped to “Greater.” Thus, the trials that produced the data in Figure 6A are comparable to Figure 5A in Experiment 1, but trials in Figure 6A were presented in the context of an overall trial structure in which the colors in hotspots were biased to map to “Greater.” We analyzed the data for the balanced cue images and hotspot-more images using separate analyses. We include the data separated by legend text position and corresponding statistical results involving legend text in the Supplementary Material (Supplementary Figure S2, Supplementary Table S2).

#### Balanced cue images

We analyzed the data in Figure 6A using a four-way repeated-measures ANOVA (2 encoded mappings × 2 hotspot lightnesses × 2 color scales × 2 legend text positions). This was the same as in Experiment 1 except for no between-subject factor of testing order. In contrast with Experiment 1 (Figure 5A), the results in Figure 6A resemble the predicted pattern if there is both a dark-is-more bias and hotspot-is more bias (Figure 2C), with faster RTs for dark-more encoding for dark hotspots but not for light hotspots. These observations are supported by a main effect of encoded mapping, an encoded mapping × hotspot lightness interaction (Table 2), and follow-up comparisons within each hotspot lightness level. For dark hotspots, where the dark-is-more bias and hotspot-is-more bias were compatible, RTs were faster for dark-more encoding than light-more encoding, *F*(1, 29) = 57.79, *p* < 0.001, $\eta_{p}^{2}$ = .666. For light hotspots, where the two biases conflicted, there was no difference between dark-more and light-more encoding (*F* < 1). This suggests that the conflicting biases canceled each other out, which may indicate that the strength of the dark-is-more bias and the hotspot-is-more bias were equated in this experiment.

**Table 2.** Results of a repeated-measures ANOVA comparing encoded mapping (Mapping) × hotspot lightness (HSLightness) × color scale (Colors) × legend text position (LegText) for balanced cue images and the same analysis but without hotspot lightness for hotspot-more images (see Experiment 2 text for details). Degrees of freedom for all tests were (1, 29). *p < .05, **p <.01, ***p < .001. Results of the full analysis (including LegText) are in Supplementary Table S2.

	Balanced cue images			Reliable cue images		
Source	*F*	*p*	$\eta_{p}^{2}$	*F*	*p*	$\eta_{p}^{2}$
Mapping	30.50	***	0.513	8.92	0.006**	0.235
HSLightness	3.27	0.081	0.101			
Colors	4.85	0.036*	0.143	4.83	0.036*	0.143
Mapping * HSLightness	31.04	***	0.517			
Mapping *Colors	0.69	0.412	0.023	0.59	0.447	0.020
HSLightness * Colors	1.04	0.317	0.035			
Mapping * HSLightness * Colors	0.20	0.662	0.007			

Why did hotspot lightness interact with encoded mapping in Experiment 2 when there was no such interaction in Experiment 1? This difference can be explained by whether hotspots were a reliable cue to the locus of larger quantities in the global structure of the experiment. In Experiment 1, hotspots were an unreliable cue over all trials. In Experiment 2, hotspots were an unreliable cue within the balanced cue image trials reported on above, but those trials were embedded in a global experiment structure in which hotspots were a reliable cue to larger quantities.

As in Experiment 1, there was an effect of color scale with faster RTs for Autumn than Viridis, but unlike Experiment 2, color scale did not interact with encoded mapping (Table 2). Comparing Autumn and Viridis in Figure 6A, there may be a tendency for the hotspot-is-more bias to dominate the dark-is-more bias for Viridis (i.e., faster RTs for light-more encoding for light hotspots), but there was not a significant three-way interaction between encoded mapping, hotspot lightness, and color scale. We return to this issue in Experiments 3 and 4.

#### Hotspot-more images

We analyzed the data for the hotspot-more images using a three-way repeated-measures ANOVA (2 encoded mappings × 2 color scales × 2 legend text positions); see Table 2. Hotspot lightness was not included as a factor because it was redundant with encoded mapping (i.e., dark-more encoding meant the hotspot was dark, and light-more encoding meant the hotspot was light). As shown in Figure 6B and supported by a main effect of encoded mapping, RTs were faster when hotspots were dark with dark-more encoding than when they were light with light-more encoding. Therefore, it was easier to interpret colormaps when hotspots were dark than when they were light, even when the hotspot mapped to larger quantities for both sets of images and the hotspot was a reliable cue. Again, RTs were faster for Autumn than Viridis.

#### Hotspot localization

In the hotspot localization task, participants saw each colormap image (without a legend) and indicated which side had the hotspot (left or right). We coded responses for each trial such that 1 = correct and 0 = incorrect and averaged over all trials for all participants to determine the overall accuracy. The mean was 0.89, indicating that participants could perceive the locations of the hotspots. We also examined mean accuracy for each of the 20 underlying data sets (averaging over color scale, hotspot side, and exact replications). The range was from 0.86 to 0.92, suggesting that participants could identify the hotspot for all colormap images.

**Figure 7.**
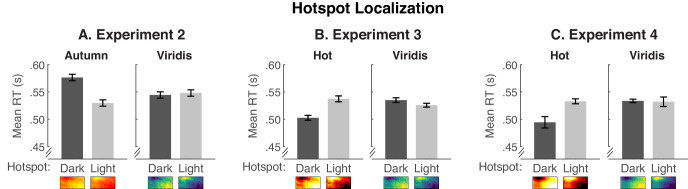
Mean RTs for the hotspot localization task in (A) Experiment 2, (B) Experiment 3, and (C) Experiment 4. Data are plotted separately for Autumn and Viridis in Experiment 2 and for Hot and Viridis in Experiments 3 and 4 for dark hotspots (dark gray bars) and light hotspots (light gray bars). Error bars represent standard errors of the means using the Cousineau (2005) adjustment to account for overall differences in RT at the subject level.

We then examined the possibility that hotspots were easier to detect in colormaps using Viridis than Autumn, given that Viridis has greater lightness and hue contrast. We pruned the RTs for hotspot localization using the same pruning procedure as before but averaged the remaining data over colormaps, left/right positions of the hotspot, and exact replications in one step. The mean RTs are shown in Figure 7A, separated by color scale and by hotspot lightness.

We analyzed the data using a two-way repeated-measures ANOVA (2 color scales × 2 hotspot lightnesses). There was no effect of color scale, *F*(1, 29) = 1.17, *p* = 0.287, $\eta_{p}^{2}$ = .039, but there was an effect of hotspot lightness, *F*(1, 29) = 7.60, *p* = 0.010, $\eta_{p}^{2}$ = .208, and a color scale × hotspot lightness interaction, *F*(1, 29) = 17.36, *p* < 0.001, $\eta_{p}^{2}$ = .375. Follow-up comparisons of hotspot lightness within each color scale indicated that participants took longer to detect dark hotspots than light hotspots for Autumn, *F*(1, 29) = 22.09, *p* < 0.001, $\eta_{p}^{2}$ = .432, whereas there was no significant difference for Viridis (*F* < 1). Thus, lower contrast in the Autumn color scale may have made hotspots harder to detect but only when hotspots were dark. Based on the results of this experiment, one might conclude that it is easiest to interpret colormaps when hotspots are dark and the legend specifies dark-more encoding (Figure 6), but it is harder to detect hotspots when they are dark (Figure 7A). However, as we will see in Experiment 3, the challenge in detecting dark hotspots is mitigated when the color scale has higher contrast.

In summary, Experiment 2 provides evidence for both a dark-is-more bias and a hotspot-is-more bias when hotspots were a reliable cue to the locus of larger quantities. RTs were faster for dark-more encoding when the hotspot was dark, but this difference diminished when the hotspot was light, suggesting these biases work together when the hotspot is dark but cancel each other when the hotspot is light. Still, for the hotspot-more images, where the legend always specified the hotspot was more, responses were reliably faster when the hotspot was dark. These results suggest that color-based biases still influence interpretability, even when there are clear spatial cues to “more.”

This experiment also demonstrated that hotspots were harder to detect for the Autumn color scale when hotspots were dark. It is possible that hotspots would have a greater dampening effect on the dark-is-more bias if the hotspots were easier to detect. We tested this hypothesis in Experiment 3 by replacing the Autumn color scale with the higher-contrast “Hot” color scale.

## Experiment 3

This experiment examined whether making hotspots more visually pronounced in colormaps would reduce the dark-is-more bias when it conflicted with the hotspot-is-more bias. We made the hotspots more pronounced by replacing the Autumn color scale (red to yellow) with the “Hot” color scale that had more lightness contrast (Figure 4). The hot color scale is similar to Autumn but is appended with the endpoints of black and white, such that it varies from black to red to yellow to white. Otherwise, Experiment 3 was identical to Experiment 2.

### Methods

#### Participants

The participants were 35 undergraduates at the University of Wisconsin–Madison (mean age = 19.34, 25 females, 10 males) who participated for extra credit in their introductory psychology course. We analyzed data from 30 participants, excluding 4 whose overall accuracy was less than 90% and 1 who did not complete the experiment. All had normal color vision and gave informed consent.

#### Design, displays, and procedure

The design, displays, and procedure were the same as Experiment 2, except the color scales were Hot and Viridis instead of Autumn and Viridis (Figure 4).

### Results and discussion

**Figure 8.**
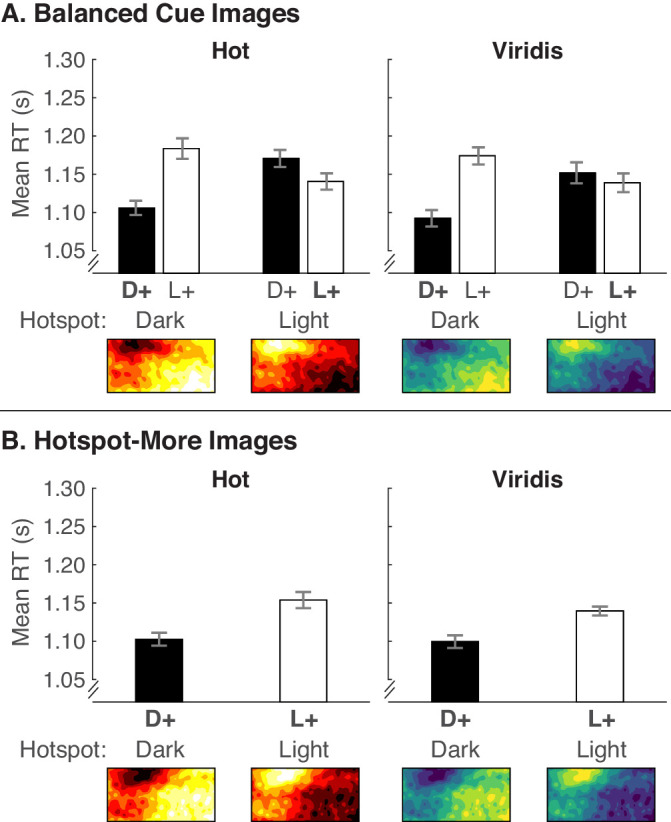
Mean RTs from Experiment 3 for (A) balanced cue images and (B) hotspot-more images, plotted in the same manner as in Figure 6.

We prepared RTs for analysis as in Experiments 1 to 2 and analyzed the data in Figure 8 using the same repeated-measures ANOVAs as in Experiment 2. We include the data separated by legend text position and corresponding statistical results involving legend text in the Supplementary Material (Supplementary Figure S3, Supplementary Table S3).

#### Balanced cue images

We predicted that replacing Autumn (lower contrast) with Hot (higher contrast) in this experiment would lead to faster RTs for dark-more encoding for dark hotspots and faster RTs for light-more encoding for light hotspots (Figure 2B). If so, that would indicate that increasing lightness contrast between the hotspot and the surround would make the hotspot-is-more bias strong enough to override the dark-is-more bias.

As shown in Figure 8A and Table 3, encoded mapping did indeed interact with hotspot lightness. Separate analyses for each hotspot lightness indicated that RTs were faster for dark-more encoded mapping for dark hotspots, *F*(1, 29) = 34.79, *p* < 0.001, $\eta_{p}^{2}$ = .545, and RTs tended to be faster for light-more encoded mapping for light hotspots, but this difference was not significant, *F*(1, 29) = 3.47, *p* = 0.073, $\eta_{p}^{2}$ = .107. Thus, these results are similar to Experiment 2. From the pattern in Figure 8A, it appeared that there might be a greater tendency for the hotspot-is-more bias to override the dark-is-more bias for Hot than for Viridis, but this three-way interaction was not significant (Table 3). Thus, making hotspots more pronounced by increasing contrast in the color scale was not sufficient to significantly override the dark-is-more bias when hotspots were light. Unlike Experiments 1 and 2, there were no effects involving color scale, now that we replaced Autumn with Hot, likely because Hot is better matched with Viridis in terms of color variability.

**Table 3.** Results of a repeated-measures ANOVA comparing encoded mapping (Mapping) × hotspot lightness (HSLightness) × color scale (Colors) × legend text position (LegText) for balanced cue images and the same analysis but without hotspot lightness for hotspot-more images (Experiment 3). Degrees of freedom for all tests were (1, 29). **p < .01, ***p < .001. Results of the full analysis (including LegText) are in Supplementary Table S3.

	Balanced cue images			Reliable cue images		
Source	*F*	*p*	$\eta_{p}^{2}$	*F*	*p*	$\eta_{p}^{2}$
Mapping	9.34	0.005**	0.244	14.45	0.001**	0.333
HSLightness	2.06	0.162	0.066			
Colors	2.20	0.149	0.070	0.85	0.364	0.029
Mapping * HSLightness	37.50	***	0.564			
Mapping * Colors	0.29	0.594	0.010	0.59	0.448	0.020
HSLightness * Colors	0.00	0.951	0.000			
Mapping * HSLightness * Colors	0.16	0.691	0.006			

#### Hotspot-more images

A main effect of encoded mapping indicated that RTs were faster when hotspots were dark than when they were light. There was no effect of color scale or color scale × encoded mapping interaction. As in Experiment 2, it was easier to interpret colormaps when hotspots were dark than when they were light, even when the hotspot mapped to larger quantities for both sets of images and the hotspot was a reliable cue.

#### Hotspot localization

We coded the hotspot localization data and pruned RTs in the same way as in Experiment 2. Overall accuracy was 0.95 and the range was from .93 to .96. Figure 7B shows the mean RTs for each color scale and hotspot lightness.

We conducted a mixed-design ANOVA to determine whether the hotspots were easier to detect for colormaps generated using Hot (Experiment 3) compared with Autumn (Experiment 2). The within-subject factors were color scale (“warm” vs. Viridis) and hotspot lightness (dark vs. light), and the between-subject factor was experiment (Experiment 2 vs. Experiment 3). We use “warm” to refer to Autumn in Experiment 2 and Hot in Experiment 3 in this overall ANOVA, but we compare Autumn and Hot in a separate analysis below. There were no overall effects of color scale, hotspot lightness, or experiment (*F*s < 1.18), but there were two-way interactions between color scale and experiment, *F*(1, 58) = 6.40, *p* = 0.014, $\eta_{p}^{2}$ = .099, and hotspot lightness and experiment, *F*(1, 58) = 9.16, *p* = 0.004, $\eta_{p}^{2}$ = .136, and a three-way interaction between color scale, hotspot lightness, and experiment, *F*(1, 58) = 52.86, *p* < 0.001, $\eta_{p}^{2}$ = .477.

To understand the three-way interaction, we separated the data by color scale, where the Viridis analysis compared the exact same images in Experiments 2 and 3, and the “warm” analysis compared Autumn (Experiment 2) and Hot (Experiment 3). For Viridis, there were no significant effects (all *F*s < 1.09). For Autumn/Hot, there was no effect of experiment, *F*(1, 58) = 2.03, *p* = 0.160, $\eta_{p}^{2}$ = .034, or hotspot lightness (*F* < 1), but there was an Experiment  ×  hotspot lightness interaction, *F*(1, 58) = 35.08, *p* < 0.001, $\eta_{p}^{2}$ = .377. Comparing Figure 7A and Figure 7B, it can be seen that RTs were faster for dark hotspots using the Hot color scale (Experiment 3) than the Autumn color scale (Experiment 2), *F*(1, 58) = 9.24, *p* = 0.004, $\eta_{p}^{2}$ = .137, and there was no difference for light hotspots (*F* < 1). Thus, we can conclude that by replacing Autumn with Hot in Experiment 3, we did make hotspots more pronounced, at least for dark hotspots that were the most difficult to detect in Experiment 2.

We now return to the concern in Experiment 2, that although it is easier to interpret colormaps when hotspots were dark, it was harder to detect hotspots when they were dark, at least for the Autumn color scale. In Experiment 3, we see the reverse pattern for Hot (Figure 7B), where it was actually easier to find hotspots when they were dark than when they were light, *F*(1, 29) = 13.37, *p* = 0.001, $\eta_{p}^{2}$ = .315. This means that it was easier to find hotspots in colormaps that were easier to interpret.

The goal of this experiment was to determine whether the hotspot-is-more bias would override the dark-is-more bias if we made hotspots more pronounced by using a color scale with greater contrast (i.e., Hot instead of Autumn). When the hotspot was dark, both biases could work together, so the critical test was to compare encoded mappings when hotspots were light and the biases conflicted. There was a nonsignificant trend for the hotspot-is-more bias to dominate the dark-is-more bias, as revealed by marginally faster RTs for light-more encoding than dark-more encoding when the hotspot was light. Still, for the hotspot-more images, responses were reliably faster when the hotspot was dark, which suggests a benefit of using dark-more encoding with dark hotspots for hotspot data visualizations.

## Experiment 4

In Experiment 3, we found a trend for the hotspot-is-more bias to override the dark-is-more bias when hotspots were light, but this difference was not significant. In Experiment 4, we attempted to further bolster the effect of the hotspot-is-more bias by making the hotspot even more pronounced. We did so by reducing the noise used to produce the hotspot images, so the location of the hotspot was even more distinct (Figure 4). Otherwise, Experiment 4 was identical to Experiment 3.

### Methods

#### Participants

The participants were 35 undergraduates at the University of Wisconsin–Madison (mean age = 18.62, 23 females, 12 males) who participated for extra credit in their introductory psychology course. We analyzed data from 30 participants, excluding 4 whose overall accuracy was less than 90% and 1 who was color deficient. All the other participants had normal color vision, and all gave informed consent.

#### Design, displays, and procedure

The design, displays, and procedure were the same as in Experiment 3, except we regenerated the underlying data sets such that they had less noise (Figure 4). We reduced the noise strength parameter for generating the Gaussian noise from 8 to 5 (see https://github.com/SchlossVRL/colormaps). We created the colormap images from the first 20 underlying data sets generated by the algorithm.

### Results and discussion

We prepared RTs for analysis using the same procedure as in Experiments 1 to 3 and analyzed the data using the same repeated-measures ANOVAs as in Experiments 2 and 3. We include the data separated by legend text position and corresponding statistical results involving legend text in the Supplementary Material (Supplementary Figure S4, Supplementary Table S4).

#### Balanced cue images

**Figure 9.**
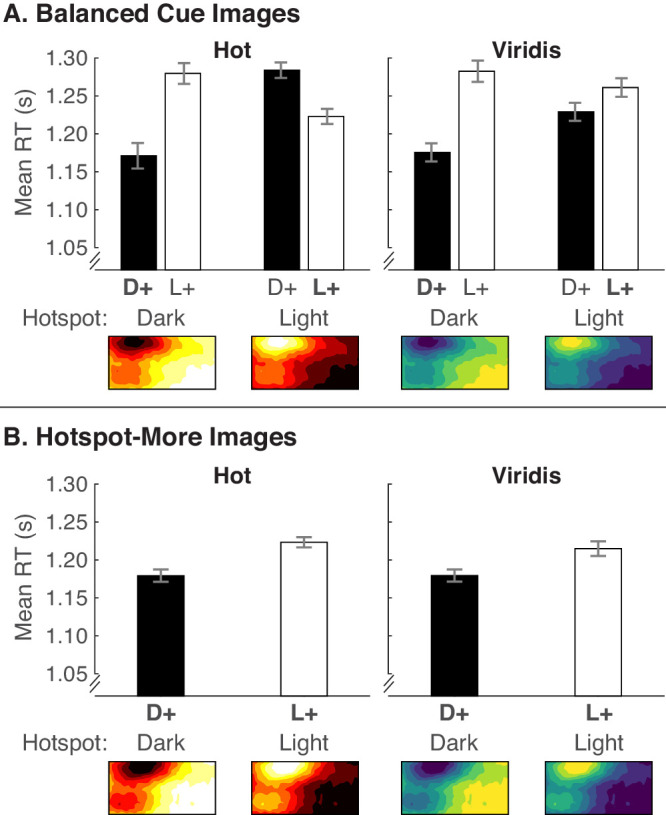
Mean RTs from Experiment 4 for (A) balanced cue images and (B) hotspot-more images, plotted in the same manner as in Figures 6 and 8.

The results of this experiment provide the first evidence that a hotspot-is-more bias can override the dark-is-more bias but only for the Hot color scale. As shown in Figure 9A, the pattern for the Hot color scale resembles the hotspot-only prediction (Figure 2B), and the pattern for the Viridis color scale resembles the dark-is-more and hotspot-is-more prediction (Figure 2C, similar also to Experiments 2 and 3). This difference in patterns between color scales is supported by a three-way interaction of encoded mapping × hotspot lightness × color scale (Table 4). Therefore, we broke down the analyses by hotspot lightness as in Experiments 2 and 3 but also examined effects involving color scale.

**Table 4.** Results of a repeated-measures ANOVA comparing encoded mapping (Mapping) × hotspot lightness (HSLightness) × color scale (Colors) × legend text position (LegText) for balanced cue images and the same analysis but without hotspot lightness for hotspot-more images (Experiment 4). Degrees of freedom for all tests were (1, 29). *p < .05, **p <.01, ***p < .001. Results of the full analysis (including LegText) are in Supplementary Table S4.

	Balanced cue images			Reliable cue images		
Source	*F*	*p*	$\eta_{p}^{2}$	*F*	*p*	$\eta_{p}^{2}$
Mapping	16.56	***	0.364	13.38	0.001**	0.316
HSLightness	4.25	0.048*	0.128			
Colors	0.05	0.831	0.002	0.19	0.664	0.007
Mapping * HSLightness	45.54	***	0.611			
Mapping * Colors	6.17	0.019*	0.176	0.30	0.587	0.010
HSLightness * Colors	0.97	0.333	0.032			
Mapping * HSLightness * Colors	6.63	0.015*	0.186			

When hotspots were dark, RTs were faster for dark-more encoded mapping, *F*(1, 29) = 37.89, *p* < 0.001, $\eta_{p}^{2}$ = .566, and encoded mapping did not interact with color scale (*F* < 1). When hotspots were light, there was no main effect of encoded mapping, *F*(1, 29) = 1.74, *p* = 0.197, $\eta_{p}^{2}$ = .057, but encoded mapping interacted with color scale, *F*(1, 29) = 15.92, *p* < 0.001, $\eta_{p}^{2}$ = .354. Thus, within the light hotspot condition, we further compared encoded mappings within each color scale. For Hot, RTs were significantly faster for *light-more encoding* than dark-more encoding, *F*(1, 29) = 17.46, *p* < 0.001, $\eta_{p}^{2}$ = .376, but for Viridis, there was no significant difference, *F*(1, 29) = 3.42, *p* = 0.075, $\eta_{p}^{2}$ = .106.

The difference between Hot and Viridis may be due to hotspots being more pronounced in colormaps constructed with the Hot color scale, given that Hot has greater lightness contrast (black to white) than Viridis (dark blue to light yellow, where dark blue is lighter than black, and light yellow is darker than white). These results suggest that under the condition in which the hotspot was most pronounced—highest color contrast and low noise in the image—the hotspot-is-more bias overrode the dark-is-more bias.

#### Hotspot-more images

When comparing cases in which the hotspot always represented larger quantities, RTs were faster for dark-more encoding than light-more encoding, and there was no interaction with color scale (Figure 9B, Table 4). This means that even though the hotspot-is-more bias dominated the dark-is-more bias for Hot (Figure 9A), there is still is a benefit of having dark hotspots over light hotspots when hotspots represent larger quantities.

#### Hotspot localization

We coded the hotspot localization data and RTs in the same way as in Experiments 2 and 3. Overall, accuracy was 0.97 (range: .93 to .99). To test if reducing the noise in the images in Experiment 4 made the hotspots easier to detect, we conducted a three-way mixed-design ANOVA comparing 2 color scales (Hot vs. Viridis) × 2 hotspots lightnesses (light vs. dark) × 2 experiments (Experiment 3 vs. Experiment 4). None of the effects involving experiment were significant (all *F*s < 1.67), suggesting reducing the noise in the images did not affect hotspot detection. This may be because hotspots were already extremely easy to detect in Experiment 3, so there was no added benefit of reducing noise. In future work, it would be helpful to systematically vary the spatial structure that determines hotspot salience within a given experiment to further understand how hotspot salience influences interpretations of colormaps.

In summary, the results of Experiment 4 suggest that there are conditions under which the hotspot-is-more bias can override the dark-is-more bias. When hotspots are light and especially pronounced (high contrast, low noise), RTs were faster for light-more encoding than dark-more encoding. Still, for the hotspot-more images, responses were reliably faster when the hotspot was dark, which further supports there being a benefit of designing hotspot data visualizations to have dark hotspots with dark-more encoding.

## General discussion

The goal of this study was to examine how people's interpretations of colormap data visualizations are influenced by color and spatial structure. Previous studies have demonstrated evidence for a dark-is-more bias, in which people infer that darker colors map to larger quantities (Cuff, 1973; McGranaghan, 1989; Schloss et al., 2019). However, it was unclear how those results would generalize to colormaps with strong data spatial structure, such as hotspots typically found in weather maps or neuroimaging brain maps. It has been proposed that when there are hotspots, people would readily infer that the largest quantities are in the center of the hotspot (Schott, 2010), which we call a hotspot-is-more bias.

We investigated whether a hotspot-is-more bias overrides the dark-is-more bias. In Experiment 1, we were surprised to find that the dark-is-more bias dominated any potential hotspot-is-more bias. In Experiments 2 to 4, we found that a hotspot-is-more bias emerged when we made hotspots a more reliable cue to “more” in the trial structure and made the hotspots more perceptually pronounced. Still, RTs were faster for dark-more encoding with dark hotspots than light-more encoding with light hotspots. Thus, the results suggest that for hotspot colormaps, it is easier to interpret the data when the hotspot is dark.

This study has furthered the field's understanding of the nature of inferred mappings for colormap visualizations, but several questions remain. Below, we highlight these questions, many of which were also raised in Schloss et al. (2019).

### Cue reliability

Would the hotspot-is-more bias entirely dominate the dark-is-more bias if the hotspot was a 100% reliable cue to the locus of larger quantities? We did not test this condition because it would have changed the nature of the task. Participants would probably have learned that the hotspot was a reliable cue in the experiment paradigm and ignored the legend. If so, the task would have been more like the hotspot localization task than the colormap interpretation task. To address this question, it would be necessary to use a different paradigm.

### Impact of robust biases that produce small RT differences

Do RT differences on the order of 100 ms have meaningful impacts on people's ability to interpret information visualizations in real-life settings? Although many of the results we report in the present study have medium to large effect sizes, we note that the differences in RTs between conditions are relatively small (∼100 ms). Some may argue that such differences are inconsequential in the grand scheme of interpreting information visualizations, especially under unlimited viewing conditions.

We have two points in response to this critique. From a theoretical perspective, the results reveal reliable and reproducible biases people have for mapping perceptual and conceptual structure, which leads to interesting questions about how such biases are formed and why they exist. From a practical perspective, the ∼100-ms differences reported here, where participants were highly concentrated and motivated to perform well, might have larger consequences under natural conditions where there are more distractions. For instance, imagine sitting in a lecture, and just as the speaker presents the colormap visualization with the critical data, you drop your pen. While picking up your pen, you get a glimpse of your phone and see that you received a text message from a loved one. You then regroup from your distractions in time to get a glimpse of the data while also trying to listen to the speaker's description of the results before the next slide appears. You had only a brief moment to process the data visualization. Whether you extracted the meaningful pattern or not may hinge on whether the encoded mapping matched your expectations, or you had to do extra work to decipher the legend. Exploring how laboratory findings scale to real-world viewing conditions is an interesting avenue for future work.

### Origins of inferred mappings

Where do inferred mappings come from, and why do people have a dark-is-more bias? Smith and Sera (1992) suggested mappings between darkness and “more” could be based in sensory systems, given that young children exhibit a dark-is-more bias. However, they also report that adults do not show a reliable dark-is-more bias and suggest this developmental change is due to exposure to unsystematic mappings between darkness and magnitude in language. Although it may seem contradictory that Smith and Sera (1992) did not find a reliable dark-is-more bias in adults, when we and others before us did (Cuff, 1973; McGranaghan, 1989; Schloss et al., 2019), the types of tasks in these studies were qualitatively different. Future work is needed to reconcile these differences and to understand the origins and developmental trajectory of the dark-is-more bias.

Some have also argued that the dark-is-more bias stems from ecological exposure to real-world objects such as increased ink on a page or other forms of density (Cuff, 1974; McGranaghan, 1989). Relatedly, the dark-is-more bias may stem from exposure to colormap data visualizations with dark-more encoding, given that it was established as a convention in statistics in the early 1900s (Palsky, 1999). As discussed in Schloss et al. (2019), studies with populations who have not had exposure to colormap data visualization will be essential for assessing the role of prior exposure to colormaps on the dark-is-more bias.

### Effects of semantic context

How might the dark-is-more bias vary with semantic context? Efforts have been under way to design colormaps to fit different types of data (e.g., data about water vs. foliage) (Samsel, Klaassen, & Rogers, 2018; Samsel, Turton, Wolfram, & Bujack, 2017), and it is possible that the dark-is-more bias might be reduced or reversed for data about content that is inherently light (e.g., sunshine). It is also possible that the dark-is-more bias might be influenced by which properties of the data are of interest. For example, if a colormap represents response time data and the focus is on which response times are fastest, people may infer that darker colors map to faster response times (i.e., smaller amounts of time). Research is currently in progress to test these possibilities.

### Effects of expertise

How does domain expertise influence inferred mappings? People's knowledge about types of visualizations and knowledge about content in visualizations can influence how they interpret data visualizations (for a review, see Shah & Hoeffner, 2002). In the present study, we chose to represent data about alien animal sightings to prevent participants from having prior knowledge about the content of the visualization. Moreover, we tested college undergraduates who have general knowledge about colormaps (e.g., from reading weather maps) but are likely not expert colormap readers. However, it is possible that domain experts, with knowledge about the (a) content in colormaps and (b) typical patterns of data in their field, may rely more on spatial structure in their data (e.g., hotspots) than inferred color-quantity mappings when interpreting colormaps. It is also possible that domain experts who are used to looking at colormaps with light-more encoded mappings (e.g., neuroscientists; see Christen et al., 2013) have developed a light-is-more bias instead of a dark-is-more bias. If so, it would be interesting to determine whether that light-is-more bias is domain specific to their area of expertise (e.g., neuroimaging) or extends across domains outside of their area of expertise (e.g., alien animal sighting maps). If one designs colormaps that align with the results of the present study but violate conventions in a particular field, there is a risk that the encoded mapping will contradict the inferred mappings of domain experts, and that could make colormaps harder to interpret.

This research is part of a larger effort to understand what factors determine correspondence between perceptual and conceptual properties in information visualizations. We have demonstrated that color and spatial factors interacted in interesting ways to determine people's inferred mappings for colormap data visualizations, and color-based inferred mapping (i.e., dark-is-more bias) was surprisingly robust when it conflicted with space-based inferred mapping (i.e., hotspot-is-more bias). By understanding how people infer meaning from visual features, it will be possible to design information visualizations that are more effective and efficient for visual communication.

## Supplementary Material

Supplement 1

## Footnotes

1 We used device-dependent RGB coordinates to specify the colormaps because we aimed to study colormaps that people typically use in real data visualizations, which are specified using RGB coordinates in software like MATLAB or packages like Matplotlib and displayed on people's own uncalibrated monitors (Schloss et al., 2019). This approach contrasts with classic color perception studies, in which the goal is to produce color specified in a device-independent space such that other laboratories can produce the exact same colors on their monitors.
