# Supplementary material for: The relation between color and spatial structure for interpreting colormap data visualizations
Source: J Vis. 2020 Nov 17;20(12):7. doi: 10.1167/jov.20.12.7 (PMC7683863; doi:10.1167/jov.20.12.7)
Supplement: Supplement 1 [file jovi-20-12-7_s001.pdf]

The relation between color and spatial structure for interpreting colormap data visualizations

SUPPLEMENTARY MATERIAL

Shannon Sibrel[1,2], Ragini Rathore[2,3], Laurent Lessard[4], and Karen B. Schloss[1,2]

[1]Department of Psychology, University of Wisconsin–Madison
[2]Wisconsin Institute for Discovery, University of Wisconsin–Madison
[3]Department of Computer Sciences, University of Wisconsin–Madison
[4]Department of Mechanical and Industrial Engineering, Northeastern University

In the main text we focused on results involving only our main factors of interest: encoded mapping, hotspot lightness, and color scale. Here we include the full sets of analyses in Tables S1-S4, which include the results reported in the main text (Tables 1-4), plus results involving legend text position for all four experiments and testing order for Experiment 1. We also show the data separated by legend text position in Figures S1-S4.

During the experiments, we balanced legend text position (i.e., "Greater" was at the top or bottom of the legend) to ensure that participants had to interpret the legend on every trial. They could not assume that the color that mapped to larger quantities was always at the top of the legend. Previous work suggested that when "Greater" was at the top of the legend, RTs were overall faster, and there were larger differences in RTs between dark-more and light-more encoding (Schloss et al., 2019). These results were consistent with the general claim that larger quantities should be represented higher in space (Hegarty, 2011; Tversky, 2011). In all four experiments in the present study, we also found robust main effects of legend text, with faster RTs when "Greater" was at the top of the legend (Tables S1-S4). In Experiment 1 we also found interactions between legend text position and encoded mapping, with a larger difference between dark-more and light-more encoded mapping when "Greater" was at the top of the legend than at the bottom (similar to Schloss et al., 2019). This interaction was not significant for the subsequent experiments when the hotspot was more dominant. In some experiments there were also higher order interactions, but they were not expected, and we do not have a theoretical account to explain them.

In Experiment 1 we counterbalanced block order for the hotspot and scrambled configuration. For both configurations, there was a main effect of order, with faster RTs for participants who completed trials for that configuration in the second block. This difference can be explained by participants having had more practice with the task when they completed the second block. For the scrambled images there were additional interactions, but the reasons for these are unclear. Block order interacted with encoded mapping, such that the degree to which RTs were faster for dark-more encoded mapping was larger when scrambled configuration trials were in the first block. Block order interacted with legend text, such that the degree to which RTs were faster when "Greater" was at the top of the legend was larger for participants who completed the scrambled configuration trials in the second block. There was also a 4-way interaction between block order, encoded mapping, legend text, and scrambled hotspot lightness.

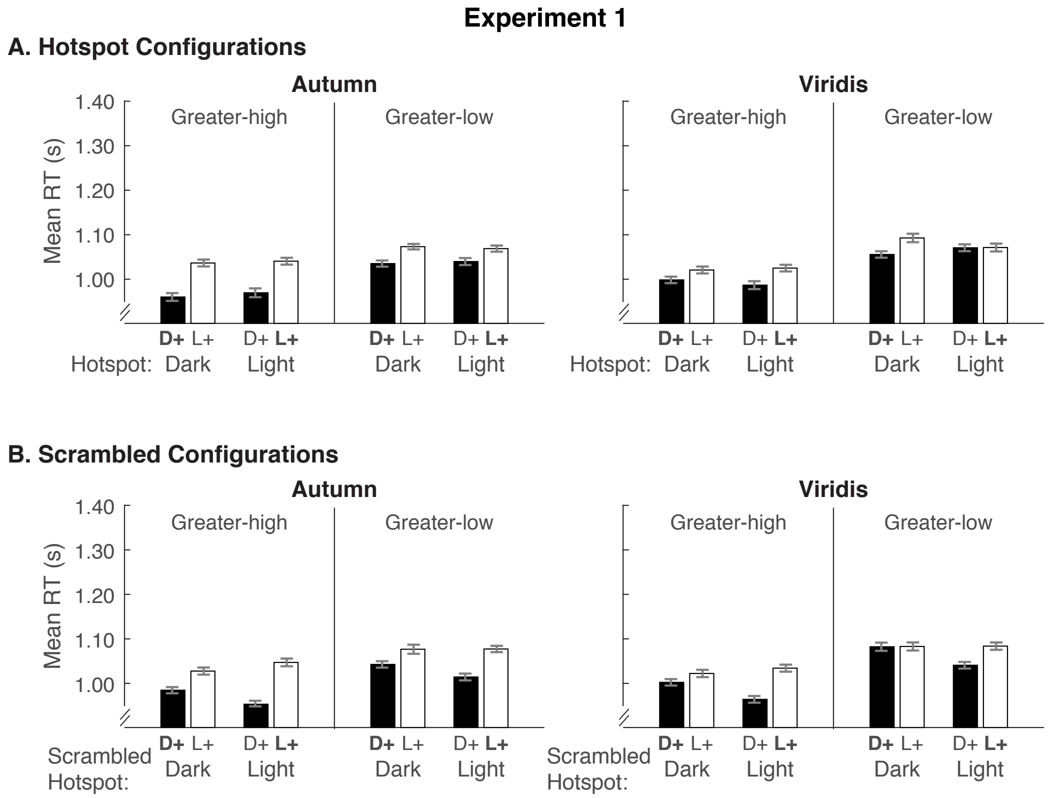

Figure S1. Mean RTs in Experiment 1 for (A) hotspot configurations and (B) scrambled configurations, separating the data by legend text position (Greater-high or Greater-low), encoded mapping (dark-more encoding, D+, black bars or light-more encoding, L+, white bars), hotspot lightness (dark or light) and color scale (Autumn or Viridis). Conditions in which the colors in the hotspot map to larger quantities are indicated by bold text (i.e., **D+** when the hotspots are dark and **L+** when hotspots are light). Error bars represent standard errors of the means using the Cousineau (2005) adjustment to account for overall differences in RT at the subject level.

Table S1. Results of a mixed-design ANOVA comparing encoded mapping (Mapping) × hotspot lightness (HSLightness) × color scale (Colors) × legend text position (LegText) × testing order (Order) for hotspot configurations and scrambled configurations (Experiment 1). Degrees of freedom for all tests were (1,58). **p* < .05, ***p* <.01, ****p* < .001.

	Hotspot Configurations			Scrambled Configurations		
	F	***p***	η_p^2	***F***	***p***	η_p^2
Mapping	36.48	***	0.386	55.99	***	0.491
HSLightness	0.00	0.993	0.000	14.39	***	0.199
Colors	10.31	0.002**	0.151	9.46	0.003**	0.140
LegText	45.62	***	0.440	47.00	***	0.448
Order	7.97	0.007**	0.121	16.69	***	0.223
Mapping * HSLightness	1.96	0.167	0.033	36.34	***	0.385
Mapping * Colors	16.80	***	0.225	8.99	0.004**	0.134
Mapping * Order	0.11	0.745	0.002	7.31	0.009**	0.112
Mapping * LegText	10.86	0.002**	0.158	7.17	0.010*	0.110
HSLightness * Colors	1.27	0.264	0.021	1.17	0.284	0.020
HSLightness * LegText	0.21	0.648	0.004	2.01	0.161	0.034
HSLightness * Order	3.97	0.051	0.064	1.37	0.247	0.023
Colors * LegText	2.82	0.099	0.046	8.47	0.005**	0.127
Colors * Order	2.49	0.12	0.041	1.71	0.196	0.029
LegText * Order	0.91	0.344	0.015	4.34	0.042*	0.070
Mapping * HSLightness * Colors	0.08	0.776	0.001	0.21	0.649	0.004
Mapping * HSLightness * LegText	6.64	0.013*	0.103	1.58	0.214	0.027
Mapping * HSLightness * Order	2.56	0.115	0.042	1.22	0.275	0.021
Mapping * Colors * LegText	6.35	0.015*	0.099	0.09	0.763	0.002
Mapping * Colors * Order	1.18	0.283	0.020	0.03	0.871	0.000
Mapping * LegText * Order	0.06	0.805	0.001	0.99	0.325	0.017
HSLightness * Colors * LegText	0.27	0.605	0.005	0.00	0.962	0.000
HSLightness * Colors * Order	0.25	0.616	0.004	2.31	0.134	0.038
HSLightness * LegText * Order	0.40	0.531	0.007	0.78	0.380	0.013
Colors * LegText * Order	0.79	0.378	0.013	0.31	0.578	0.005
Mapping * HSLightness * Colors * LegText	4.72	0.034*	0.075	0.34	0.562	0.006
Mapping * HSLightness * Colors * Order	1.44	0.236	0.024	1.22	0.273	0.021
Mapping * HSLightness * LegText * Order	0.93	0.338	0.016	5.73	0.020*	0.090
Mapping * Colors * LegText * Order	0.12	0.726	0.002	0.74	0.392	0.013
HSlightness * Colors * LegText * Order	0.01	0.946	0.000	0.10	0.751	0.002
Mapping * HSlightness * Colors * LegText * Order	1.65	0.204	0.028	0.16	0.690	0.003

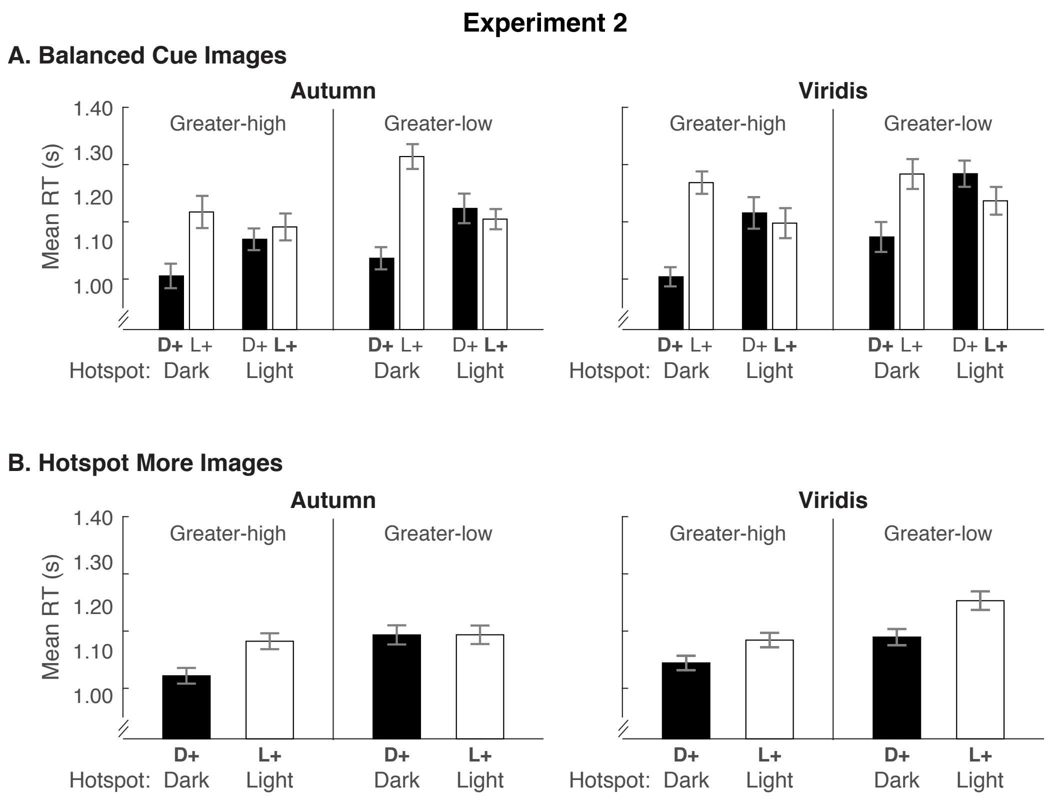

Figure S2. Mean RTs for (A) balanced cue images and (B) hotspot more images in Experiment 2 corresponding to Figure 6 of the main text, but separated by legend text position (greater-high or greater-low in the legend).

Table S2. Results of a repeated-measures ANOVA comparing encoded mapping (Mapping) × hotspot lightness (HSLightness) × color scale (Colors) × legend text position (LegText) for balanced cue images, and the same analysis but without hotspot lightness for hotspot more images (see Experiment 2 text for details). Degrees of freedom for all tests were (1,29). **p* < .05, ***p* <.01, ****p* < .001.

	**Balanced Cue Images**			**Hotspot More Images**		
	F	***p***	η_p^2	***F***	***p***	η_p^2
Mapping	30.50	***	0.513	8.92	0.006**	0.235
HSLightness	3.27	0.081	0.101			
Colors	4.85	0.036*	0.143	4.83	0.036*	0.143
LegText	15.75	***	0.352	20.09	***	0.409
Mapping * HSLightness	31.04	***	0.517			
Mapping *Colors	0.69	0.412	0.023	0.59	0.447	0.020
Mapping * LegText	0.26	0.617	0.009	1.19	0.284	0.039
HSLightness * Colors	1.04	0.317	0.035			
HSLightness * LegText	0.27	0.607	0.009			
Colors * LegText	0.00	0.973	0.000	0.52	0.479	0.017
Mapping * HSLightness * Colors	0.20	0.662	0.007			
Mapping * HSLightness * LegText	0.63	0.433	0.021			
Mapping * Colors * LegText	1.39	0.248	0.046	5.69	0.024*	0.164
HSLightness * Colors * LegText	0.68	0.415	0.023			
Mapping * HSLightness * Colors * LegText	3.06	0.091	0.095			

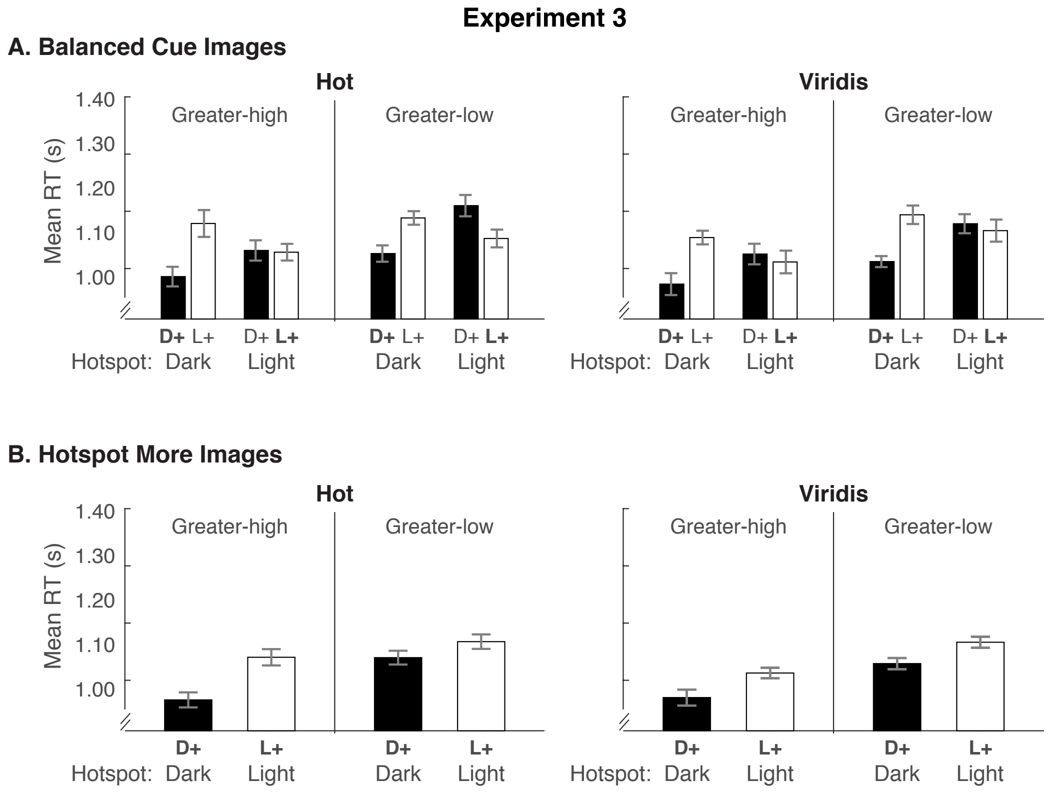

Figure S3. Mean RTs for (A) balanced cue images and (B) hotspot more images in Experiment 3 corresponding to Figure 8 of the main text, but separated by legend text position (greater-high or greater-low in the legend).

Table 3. Results of a repeated-measures ANOVA comparing encoded mapping (Mapping) × hotspot lightness (HSLightness) × color scale (Colors) × legend text position (LegText) for balanced cue images, and the same analysis but without hotspot lightness for hotspot more images (Experiment 3). Degrees of freedom for all tests were (1,29). *p < .05, **p <.01, ***p < .001.

	Balanced Cue Images			Hotspot More Images		
	F	***p***	η_p^2	***F***	***p***	η_p^2
Mapping	9.34	0.005**	0.244	14.45	0.001**	0.333
HSLightness	2.06	0.162	0.066			
Colors	2.20	0.149	0.070	0.85	0.364	0.029
LegText	13.30	0.001**	0.314	39.55	***	0.577
Mapping * HSLightness	37.50	***	0.564			
Mapping * Colors	0.29	0.594	0.010	0.59	0.448	0.020
Mapping * LegText	1.28	0.267	0.042	3.34	0.078	0.103
HSLightness * Colors	0.00	0.951	0.000			
HSLightness * LegText	1.94	0.175	0.063			
Colors * LegText	0.35	0.558	0.012	0.11	0.740	0.004
Mapping * HSLightness * Colors	0.16	0.691	0.006			
Mapping * HSLightness * LegText	0.11	0.748	0.004			
Mapping * Colors * LegText	2.23	0.146	0.071	1.23	0.276	0.041
HSLightness * Colors * LegText	0.17	0.684	0.006			
Mapping * HSLightness * Colors * LegText	0.11	0.740	0.004			

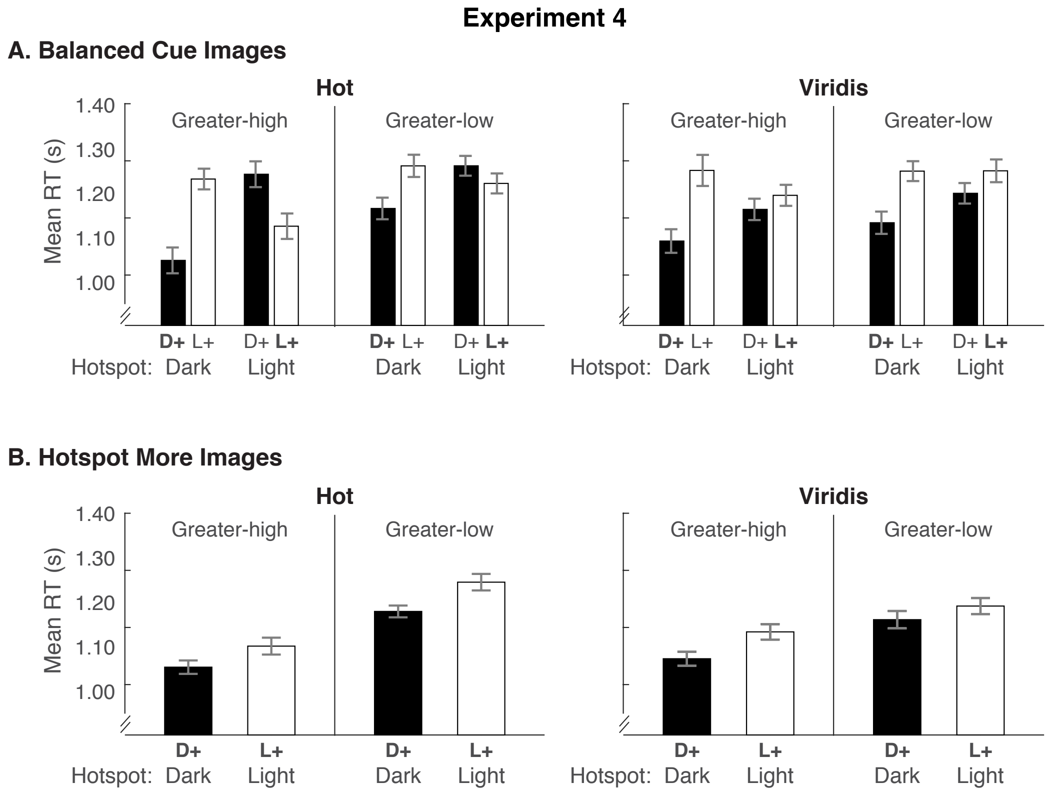

Figure S4. Mean RTs for (A) balanced cue images and (B) hotspot more images in Experiment 4 corresponding to Figure 9 of the main text, but separated by legend text position (greater-high or greater-low in the legend).

Table 4. Results of a repeated-measures ANOVA comparing encoded mapping (Mapping) × hotspot lightness (HSLightness) × color scale (Colors) × legend text position (LegText) for balanced cue images, and the same analysis but without hotspot lightness for hotspot more images (Experiment 4). Degrees of freedom for all tests were (1,29). *$p < .05$, **$p < .01$, ***$p < .001$.

	**Balanced Cue Images**			**Hotspot More Images**		
	F	***p***	η_p^2	***F***	***p***	η_p^2
Mapping	16.56	***	0.364	13.38	0.001**	0.316
HSLightness	4.25	0.048*	0.128			
Colors	0.05	0.831	0.002	0.19	0.664	0.007
LegText	8.92	0.006**	0.235	33.64	***	0.537
Mapping * HSLightness	45.54	***	0.611			
Mapping * Colors	6.17	0.019*	0.176	0.30	0.587	0.010
Mapping * LegText	0.07	0.797	0.002	0.07	0.801	0.002
HSLightness * Colors	0.97	0.333	0.032			
HSLightness * LegText	0.04	0.838	0.001			
Colors * LegText	1.24	0.274	0.041	5.74	0.023*	0.165
Mapping * HSLightness * Colors	6.63	0.015*	0.186			
Mapping * HSLightness * LegText	5.42	0.027*	0.157			
Mapping * Colors * LegText	0.02	0.897	0.001	1.09	0.305	0.036
HSLightness * Colors * LegText	0.56	0.461	0.019			
Mapping * HSLightness * Colors * LegText	1.02	0.321	0.034			
